# Fine-scale population structure of Malays in Peninsular Malaysia and Singapore and implications for association studies

**DOI:** 10.1186/s40246-015-0039-x

**Published:** 2015-07-22

**Authors:** Boon-Peng Hoh, Lian Deng, Mat Jusoh Julia-Ashazila, Zakaria Zuraihan, Ma’amor Nur-Hasnah, Ab Rajab Nur‐Shafawati, Wan Isa Hatin, Ismail Endom, Bin Alwi Zilfalil, Yusoff Khalid, Shuhua Xu

**Affiliations:** Chinese Academy of Sciences (CAS) Key Laboratory of Computational Biology, Max Planck Independent Research Group on Population Genomics, CAS-MPG Partner Institute for Computational Biology (PICB), Shanghai Institutes for Biological Sciences, Chinese Academy of Sciences, Shanghai, 200031 China; Institute of Medical Molecular Biotechnology, Faculty of Medicine, Universiti Teknologi MARA, Sungai Buloh Campus, Selangor, Malaysia; UCSI University, Kuala Lumpur Campus, Cheras, Kuala Lumpur, Malaysia; Human Genome Centre, School of Medical Sciences, Universiti Sains Malaysia, Kelantan, 16150 Malaysia; Faculty of Science and Technology, School of Biosciences and Biotechnology, Universiti Kebangsaan Malaysia, Bangi, 43600 Malaysia; Department of Pediatrics, School of Medical Sciences, Universiti Sains Malaysia, Kelantan, 16150 Malaysia; UCSI University, Jalan Menara Gading, Taman Connaught, 56000 Kuala Lumpur, Wilayah Persekutuan Malaysia; School of Life Science and Technology, ShanghaiTec University, Shanghai, 200031 China; Collaborative Innovation Center of Genetics and Development, Shanghai, 200438 China

**Keywords:** Malay, Population sub-structure, F_ST_, Latitude-PC correlation, GWAS simulation

## Abstract

Fine scale population structure of Malays - the major population in Malaysia, has not been well studied. This may have important implications for both evolutionary and medical studies. Here, we investigated the population sub-structure of Malay involving 431 samples collected from all states from peninsular Malaysia and Singapore. We identified two major clusters of individuals corresponding to the north and south peninsular Malaysia. On an even finer scale, the genetic coordinates of the geographical Malay populations are in correlation with the latitudes (R^2^ = 0.3925; P = 0.029). This finding is further supported by the pairwise F_ST_ of Malay sub-populations, of which the north and south regions showed the highest differentiation (F_ST [North–south]_ = 0.0011). The collective findings therefore suggest that population sub-structure of Malays are more heterogenous than previously expected even within a small geographical region, possibly due to factors like different genetic origins, geographical isolation, could result in spurious association as demonstrated in our analysis. We suggest that cautions should be taken during the stage of study design or interpreting the association signals in disease mapping studies which are expected to be conducted in Malay population in the near future.

**Electronic supplementary material:**

The online version of this article (doi:10.1186/s40246-015-0039-x) contains supplementary material, which is available to authorized users.

## Background

Malaysia, a multi-ethnic, multi-lingual, multi-cultural and multi-religious country, is located at the crossroads of Southeast Asia. It is separated by the South China Sea into two land masses namely, the Peninsular Malaysia and East Malaysia (also known as the Borneo island). Malaysia has a total population of about 30 million people, of which approximately 26 million populate the Peninsular Malaysia. Among the major populations in Peninsular Malaysia, the Malays are the largest ethnic group and make up to 63% of the total population follow by Chinese, Indians and other minority ethnic groups.

Many Malays are of Malayo-Polynesian (Austronesian) origin that are culturally and historically heterogeneous [1]. The Malays from the west coast of Peninsular Malaysia are historically linked to Sumatera across the Straits of Malacca; while those from the south are thought to have migrated from Jawa, Sulawesi and other parts of Indonesia [2]. The Malays from the north Peninsular have a closer affinity to the Malay Muslims from the Southern Thai due to geographical location. The history of Singapore has never been separated from Peninsular Malaysia since the first century until the year 1965 when Singapore became an independent republic. Therefore, it is very likely that the Malays of Singapore have a similar history of origin as those from the southern part of Peninsular Malaysia [3].

Earlier studies had indicated potential genetic sub-structure among the different groups of Malays from Peninsular Malaysia [2, 4, 5], which could be possibly attributed to the migration history of these respective sub-groups. However, fine-scale sub-structure of the Malay population remained poorly described, especially, previous studies were based on very limited sample size. Indeed, this potentially poses confounding factors to the genetic association studies, in particular genome-wide association studies (GWAS), leading to spurious association signals [6]. Being one of the major populations in the Southeast Asia, characterizing population substructure is crucial in designing, analyzing and interpreting any genetic association study in this region.

In this study, we showed that the genetic diversity and population sub-structure of the Malays from Peninsular Malaysia are correlated to the geographical latitude. Notably, we observed the main differentiations between populations corresponding to the north and south Peninsular Malaysia. In addition, simulation analyses carried out also revealed that the genetic association is greatly affected by population sub-structure, suggesting that consideration of population stratification of samples at the stage of study design and careful interpretation of the association signals are necessary when mapping complex diseases in Malay populations.

## Results

### Population substructure

**Fig. 1:**
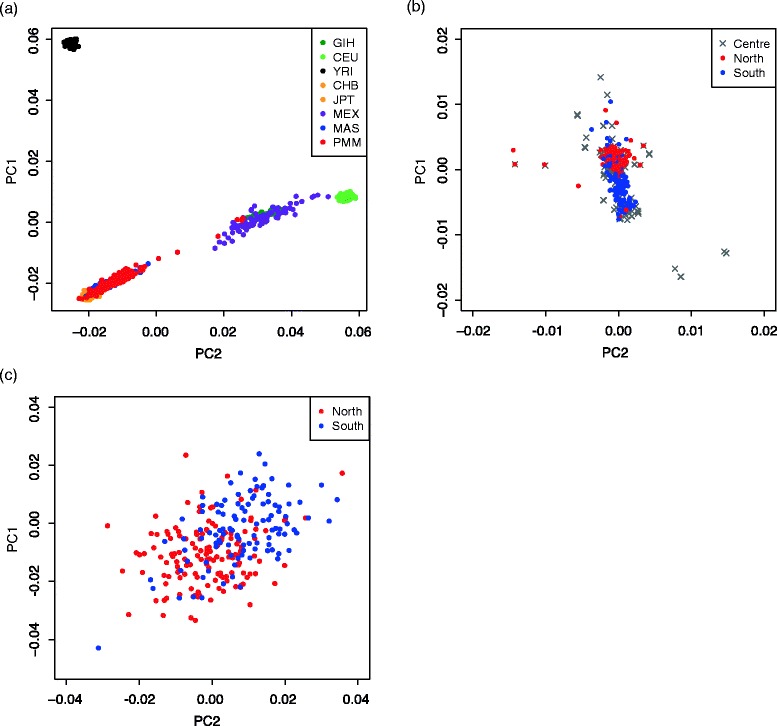
Principle Component Analysis (PCA) (**a**) Global PCA including populations from HapMap3. GIH, Gujarati India Houston; CEU, Northern and Western European from CEPH collection; YRI, Yoruba Ibadan from Nigeria; CHB, Chinese Beijing; JPT, Japanese Tokyo; MEX, Mexican ancestry from Los Angeles; MAS, Metropolitan Malays from Singapore; PMM, Malays from Peninsular Malay. The Malay populations are of East Asian descendant. (**b**) PCA plot including samples categorized into North vs Centre vs South; (**c**) PCA plot which included only North vs South. Symbols in red represent the northern region; symbols in blue represent southern region. Several outliers were excluded from the PCA plot

We first compared the genetic diversity of the Peninsular Malays from a global scale with 6 populations from HapMap3 including YRI, CHB, JPT, CEU, MEX and GIH. PC plot indicated that the Malays clustered closely to the East Asian populations as expected, and showed a rather small genetic diversity. Several Malay individuals from northern Peninsular Malaysia (PMM) showed closer affinity to the South Asia populations (GIH) (Fig. 1a). We then performed PCA for the Peninsular and Singapore Malays, and revealed a seemingly homogenous cluster (Additional file 1: Figure S1). However, some level of differentiations were observed corresponding to three geographical regions (north, center and south), despite samples from center region that was scattered around (Fig. 1b). We subsequently excluded the samples form the center regions (Pahang and Selangor), and re-ran the *smartPCA*. Two clusters were observed representing the north and south regions, respectively (Fig. 1c).

**Fig. 2:**
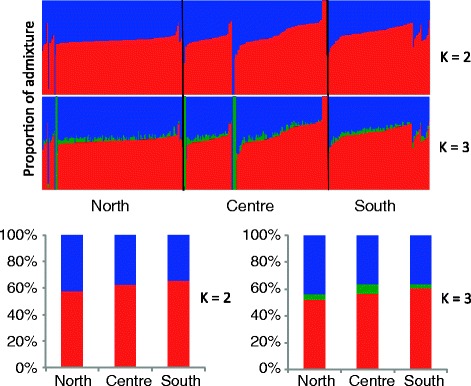
ADMXITURE analysis of the Malay populations classified according to regions. The bottom plots represented by percentages (Y-axis) indicates the average ADMIXTURE values for each region

In ADMIXTURE analysis, a significant difference was observed between the Malays from the north and south in the major component, with 57% and 65% in the north and south, respectively (P < 0.0001; Fig. 2). At K=3, the newly appeared component (denoted in green) was seen slightly higher in the central Malays than in the south Malays (6.8% vs 3%; P = 0.0415).

### Correlation of genetic and geographic coordinates

**Fig. 3:**
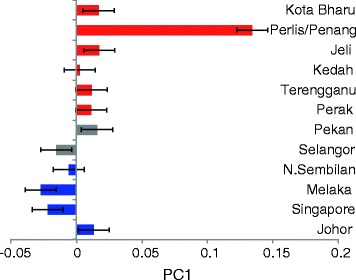
Average PC1 values of the Malay sub-populations from Peninsular Malaysia and Singapore. Standard error of each population is indicated. The PC1 values correlated well to the geographical locations of each population except for Johor

**Fig. 4:**
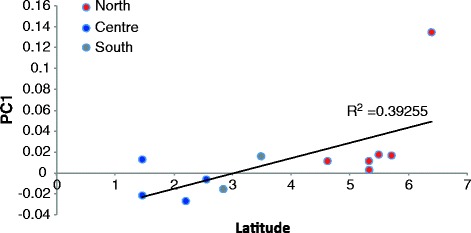
Correlation between PC1 and latitude coordinate (P = 0.029)

Given the fact that the PC1 as well as the ADMIXTURE analysis showed significant differences between northern and southern Malay samples, we then investigated if the genetic diversity between these sub-structure of Malays in Peninsular Malaysia were attributed to geographical coordinates. Average PC1 values of southern Malay samples (corresponding to Fig. 1b) were generally less than 0 (except for Johor), whilst all geographically defined northern regions with PC1 >0 (Fig. 3). When we compared the PC1 with geographical latitude of these sample locations, a significant correlation was observed (R^2^ = 0.3925; P = 0.029; Fig. 4). Due to the geographical nature, Peninsular Malaysia is divided into west coast and east coast by the Titiwangsa Ranges. We therefore asked if the genetic diversity could be attributed to the geographical longitude as well. Analysis between PC1 and geographical longitude, however showed no significant correlation (R^2^=0.0066; P = 0.7924; Addional file 1: Figure S2). We also evaluated if the genetic diversity was related to geographical distance between two populations, but found no significant correlation of F_ST_ between populations and the geographical distances between them (R^2^ = 0.01918; P = 0.1385; Additional file 1: Figure S3).

### Genetic differentiation between northern and southern Malays

The regional F_ST_ values indicated the highest regional diversity between the north and the south after 1,000 times bootstrapping repeats (F_ST_ = 0.001; CI = 2.07E - 04) (Table 1; Additional file 2: Table S1). To further identify the genomic regions that are highly differentiated between northern and southern Peninsular Malay, we computed the F_ST_ values of the 41,400 SNPs between northern and southern samples, and identified 428 SNPs listed in the top 1% of the F_ST_ (Additional file 2: Table S2); of which 80 (0.1%) had an F_ST_ value >0.05 (Table 2). SNP with the highest F_ST_ value was rs4149264, residing in the candidate gene *ABCA1* - a major gene responsible for high-density lipolipoprotein cholesterol (HDL-c) synthesis. Another highly differentiated SNP, rs4148475, is located at the candidate gene *ABCC4*. This gene is a member of the superfamily of ATP-binding Cassette (ABC) transporters, which may play a role in cellular detoxification [7]. A missense variant rs1056836 appears to be one of the four highly differentiated SNPs, leading to a change of valine to leucine in candidate gene *CYP1B1*, which had a minor allele frequency of 0.48 and 0.19 in northern and southern peninsular Malays, respectively (F_ST_ = 0.2037). This candidate gene is responsible in drug metabolism and synthesis of cholesterols, steroids and lipids. It was found to play a role in the susceptibility of glaucoma [8, 9]. We performed an enrichment analysis with DAVID (http://david.abcc.ncifcrf.gov/) by including the top 1% highly differentiated variants, but identified no significant enrichment after Benjamini correction (Additional file 2: Table S3).

**Table 1:** Pairwise F_ST_ bootstrap values of the Malay between the 3 regions of Peninsular Malaysia

	North	Centre	South
North	-	0.00083315 (CI =2.0684E-04)	0.00111661 (CI = 2.68108E-06)
Centre		-	0.00058556 (CI = 4.0972E-06)
South			-

Pairwise F_ST_ values calculated by bootstrap resampling 1,000 replications

**Table 2:** Top 0.1 % SNPs that are highly differentiated between the Malays from northern and southern region of Peninsular (total SNP = 42633)

rsID	Chr	Position	Minor allele	F_ST_	MAF_North	MAF_South	Gene	Category
rs4149264	9	107,677,211	C	0.2256	0.4856	0.1682	*ABCA1*	intronic
rs10102377	8	83,762,822	T	0.2251	0.4097	0.2336		
rs4148475	13	95,853,574	A	0.2242	0.4676	0.184	*ABCC4*	intronic
rs1056836	2	38,298,203	G	0.2037	0.4757	0.1934	*CYP1B1*	coding
rs1126965	17	70,642,790	G	0.1931	0.5	0.1822	*SLC39A11*	3utr
rs17769090	15	70,630,120	A	0.1636	0.4648	0.2381		
rs6974363	7	47,633,187	G	0.1421	0.493	0.2333		
rs837395	1	47,269,338	A	0.1400	0.4897	0.2383	*CYP4B1*	intronic
rs4646430	2	38,306,415	G	0.1384	0.4621	0.1981		
rs215101	16	16,052,973	G	0.1206	0.4752	0.271	*ABCC1*	intronic
rs12920607	16	73,728,620	C	0.1183	0.475	0.2736		
rs837398	1	47,266,422	A	0.1124	0.4897	0.2664	*CYP4B1*	intronic
rs809367	10	89,741,806	A	0.1088	0.4307	0.2009		
rs316133	6	52,847,551	C	0.0957	0.4823	0.2594	*GSTA4*	intronic
rs6130511	20	42,681,088	A	0.0916	0.2801	0.1	*TOX2*	intronic
rs2132845	4	140,587,125	T	0.0910	0.4255	0.215	*MGST2*	5utr
rs5761313	22	26,313,745	T	0.0887	0.4964	0.2804	*MYO18B*	intronic
rs10485805	20	54,945,783	G	0.0853	0.4397	0.2336	*AURKA*	intronic
rs10489142	1	7,363,310	G	0.0835	0.4507	0.3364	*CAMTA1*	intronic
rs2274928	13	24,044,546	A	0.0779	0.3601	0.4346	*LINC00327*	intronic
rs11935505	4	145,226,422	A	0.0758	0.04643	0.1682		
rs1566869	12	52,266,348	A	0.0695	0.3406	0.1682		
rs1884897	20	6,612,832	G	0.0695	0.2812	0.1215		
rs4530975	7	104,415,415	T	0.0679	0.1862	0.05607	*LHFPL3*	intronic
rs6024831	20	54,938,464	G	0.0675	0.4161	0.3915	*FAM210B*	intronic
rs1160798	6	112,438,446	C	0.0661	0.06897	0.1934	*LAMA4*	intronic
rs2158196	4	114,416,596	C	0.0658	0.2391	0.09434	*CAMK2D*	intronic
rs16961766	13	103,899,499	A	0.0645	0.3143	0.1524		
rs10962015	9	15,387,949	A	0.0638	0.2482	0.1028		
rs6884962	5	172,682,382	A	0.0623	0.4896	0.3271		
rs17126776	12	39,311,625	A	0.0603	0.1759	0.3318		
rs2755209	13	41,137,804	C	0.0602	0.25	0.1075	*FOXO1*	intronic
rs9783586	13	108,361,559	T	0.0601	0.2671	0.4393	*FAM155A*	intronic
rs10968093	9	27,753,227	A	0.0594	0.06338	0.1776		
rs2458286	8	103,978,699	T	0.0591	0.4424	0.3774		
rs4875364	8	4,444,592	C	0.0583	0.3094	0.1557	*CSMD1*	intronic
rs11145506	9	80,264,584	T	0.0578	0.3821	0.217	*GNA14*	intronic
rs11604366	11	28,887,766	C	0.0577	0.2695	0.4387		
rs9881633	3	112,881,539	T	0.0574	0.3	0.472	*RP11_572M11.3*	intronic
rs5762448	22	28,408,444	C	0.0573	0.3514	0.1916	*TTC28*	intronic
rs6467991	7	83,954,737	C	0.0570	0.344	0.4811		
rs10089677	8	122,660,248	A	0.0569	0.2329	0.09813		
rs1923254	13	41,084,241	G	0.0567	0.3776	0.2143		
rs7813806	8	5,142,665	C	0.0567	0.2817	0.1355		
rs7625411	3	112,811,428	A	0.0564	0.3403	0.486		
rs2922249	6	127,954,614	C	0.0564	0.09441	0.2196		
rs2294088	8	124,526,607	A	0.0564	0.4514	0.2804	*FBXO32*	intronic
rs12289262	11	12,894,758	T	0.0560	0.3986	0.2336	*TEAD1*	intronic
rs4937523	11	130,347,190	T	0.0558	0.2937	0.4626	*ADAMTS15*	intronic
rs10807768	7	13,662,014	A	0.0554	0.3169	0.1651		
rs976272	14	61,449,328	A	0.0551	0.4897	0.3178	*SLC38A6*	coding
rs13027801	2	143,602,503	C	0.0551	0.2862	0.4533		
rs17701834	19	22,121,458	G	0.0549	0.2172	0.08879		
rs7193843	16	54,677,292	G	0.0548	0.1448	0.285		
rs7097885	10	16,506,501	C	0.0542	0.2832	0.4486	*PTER*	intronic
rs2791398	1	245,965,551	G	0.0540	0.05944	0.1651	*SMYD3*	intronic
rs10486802	7	39,723,768	A	0.0531	0.1884	0.07009	*RALA*	intronic
rs8031676	15	96,910,440	C	0.0529	0.4306	0.2664		
rs7186479	16	82,602,736	C	0.0527	0.2517	0.1168		
rs6054383	20	6,584,604	T	0.0526	0.3986	0.2383		
rs4460308	7	104,420,060	C	0.0521	0.1866	0.07009	*LHFPL3*	intronic
rs3775779	4	70,709,207	A	0.0520	0.476	0.3551	*SULT1E1*	intronic
rs9375877	6	132,690,239	G	0.0517	0.4862	0.3458	*MOXD1*	intronic
rs2180691	20	54,964,361	A	0.0517	0.45	0.2857	*AURKA*	intronic
rs7778955	7	39,740,487	G	0.0517	0.1438	0.04206	*RALA*	intronic
rs4608114	12	92,384,658	A	0.0517	0.4366	0.2736	*C12orf79*	intronic
rs6946733	7	106,670,288	A	0.0514	0.4281	0.2664		
rs816650	10	601,089	T	0.0514	0.114	0.2406	*DIP2C*	intronic
rs6490805	13	24,084,809	C	0.0507	0.08394	0.1981		
rs17171480	7	35,585,669	A	0.0506	0.09286	0.2103		
rs17015112	3	77,319,487	G	0.0505	0.4545	0.3785	*ROBO2*	intronic
rs573186	3	124,178,276	C	0.0503	0.2483	0.1168	*KALRN*	intronic
rs10752609	1	154,791,128	A	0.0501	0.3147	0.1698	*KCNN3*	intronic
rs1862737	16	75,281,964	C	0.0500	0.4155	0.257	*BCAR1*	5utr

We observed that 1,666 SNPs were presented in different minor alleles between the north and south Malays, and their allele frequencies in Malays were compared with that in South Asian (GIH) and East Asian (CHB) (Additional file 2). Although not substantial, differences in allele frequencies were observed between the South- and East- Asians, as well as the between the Malays and both South- and East- Asians. Notably, rs1126965 located at the candidate gene *SLC39A11* revealed an alternative allele frequency of 0.8178 in the northern Malays and 0.4965 in the southern Malays. This gene has been reported to play a role in liver enzyme and smoking initiation [10, 11]. Whether or not this gene is under positive selection in the Malays, however, remain further investigation. We subsequently assessed if these SNPs play a role in phenotypic association, and found that 19 of these SNPs were reported in GWAS catalogue (Additional file 4).

To evaluate the potential effect of population sub-structure on a disease association study, a series of computer simulation studies were carried out with PLINK following a case–control GWAS design (Additional file 2: Table S4). The GWAS simulations revealed that the effect on false positive rate and statistical power were greater than expected [12].

## Discussion

We demonstrated in this study, that the Malays from Peninsular Malaysia and Singapore are essentially sub-structured. Although genetic correlation with geographical latitude had been previously reported in the Chinese populations [12, 13], it is indeed surprising to reveal such differentiation among the Malay populations even within a small region in Peninsular Malaysia and Singapore (~800 KM from north to south). In addition to that, the F_ST_ between the north and south Malays were similar to those of the earlier report between the northern and southern Han Chinese (F_ST_ = 0.0011) [12] but lower than those within Europeans (F_ST_ = 0.0033) [14] However, we observed higher diversity within the substructures of the Malays. For instance, the F_ST_ between two northern Peninsular Malays from Kedah and Kelantan was 0.017 (Table S4), which is in line with the finding in a recent study [4]. This suggests higher heterogeneity among Malays than previously expected, possibly be due to the recent migration and gene flow from the surrounding populations in this region.

The Pahang Malays were found to have a closer affinity to the north, although they were classified as the central region in this study. This is likely due to the reason that samples were collected from the Federal Land Development Authority (FELDA) settlers in the Pahang state, of which the majority of them were originated from Kelantan. On a separate note, Selangor, being as the most advanced and most populated state of Malaysia, is where the metropolitan city Kuala Lumpur located. PCA revealed that samples from this population was scattered across both the north and south regions (Fig. 1b & c). We believe that urbanization had likely blurred the boundaries. Similar findings were observed in Xu et al. (2009), where the populations from metropolitan areas showed more complicated composition with multiple ancestral origins compared with those from the rest of the area.

Essentially, identification a panel of ancestry informative markers (AIMs) would be an ideal strategy to correct the population stratification in future genetic association studies [15]. However, the SNP coverage and the sample size in the current study are insufficient for such purpose. Those highly differentiated SNPs between the north and south Malays could be possibly due to genetic drift or, to a lesser extent, natural selection. These SNPs, however may be considered as the putative set of variants as the AIMs for the Malay populations. The candidate gene *ABCA1* is a major gene that plays an important role in high-density lipoprotein cholesterol (HDL-c) synthesis and cholesterol transport [16]. However, whilst we suspect the genetic drift is likely to be the cause, the reason of this gene being highly differentiated between northern and southern Malays remains further investigated. Cautions should be taken though when positive signals of HDL-c and *ABCA1* are identified in the genetic association study of Malays.

We acknowledge several limitations in this study. Sample collection from several locations were small, hence might have resulted into outliers which confounded the outcome of the correlation between genetic differentiation and geographical coordinates. In addition, self-reported ancestry might have also confounded the analysis when assigning to their respective state of origin. However, the number of samples covering all states in Peninsular Malaysia (and Singapore), and the marker utilized in our study are larger than the previous reports, thus provides further insights into the genetic structure of the Malays in Peninsular Malaysia. Notably, we revealed close relationship between genetic and geographical coordinates in the Malay populations. In addition, our results and to which extent the admixtures in Southeast Asia could impact the population stratification thus affect the genetic association studies. Therefore we call for attention to look into alternative strategies for disease mapping in genetically complex populations particularly from Southeast Asia.

## Conclusion

In summary, we revealed that the population substructure of the Malays was correlated to the latitude coordinate. The genetic diversity of the Malays is more heterogeneous than previously expected, and that we proved that such population sub-structure occurred even though within a small geographical region may potentially lead to spurious signals in disease based genetic association studies. Therefore cautions should be taken when carrying out such study design.

## Methods

### Population and samples

**Fig. 5:**
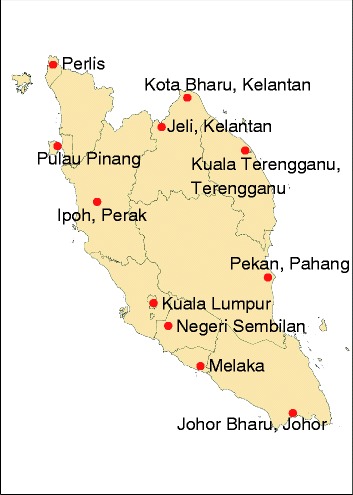
The geographical map of Peninsular Malaysia. The sampling locations are shown in red dots

A total of 431 Malay samples were included in this study. These samples were self-identified Malays from Peninsular Malaysia, 116 of which were genotyped with Affymetrix Genome-Wide Human SNP Array 6.0, whilst the remaining samples were genotyped with Illumina 660W (Sample size, N = 90) and Illumina Omni Express (N = 119). The additional 17 Malays samples from Kelantan genotyped with Affymetrix Genome-Wide Human SNP Array 6.0 [17], and 89 samples of metropolitan Malays from Singapore (SGVP) were also included in this analysis [3]. The studies were approved by the research and ethics committees of Universiti Teknologi MARA and Universiti Sains Malaysia, and the design of this study followed the Helsinki Declaration 1975, as revised in year 2000. The collected samples covered all 11 states of Peninsular Malaysia (Fig. 5), of which were divided into 3 geographical regions for the purpose of this study namely, North, South and the Centre regions, according to their respective latitude coordinate (Table 3). The number of samples and their geographical locations are listed in Table 3. Six selected populations involving 805 samples from the International HapMap Project 3 (HapMap 3) [18] were included in the analysis to characterize the genetic variation of the Malays on a global scale: YRI, GIH, CEU, CHB, JPT and MEX.

**Table 3:** Regional categorization of the Peninsular Malaysia states according to geographical locations and final number of sample included after QC

Region	States	Latitude coordinate*	No. subjects
North	Perlis	6°23′40.06″N (6.394462)	7
	Kedah	5°19′45.2″N (5.329221)	
	Pulau Pinang	5°19′45.2″N (5.329221	
	Perak	4°37′8.46″N (4.619018)	5
	Kelantan (Kota Bharu)	5°42′55.13″N (5.715314)	56
	Kelantan (Jeli)	5°29′49.4″N (5.497056)	74
	Terengganu	5°19′30.33″N (5.325092)	4
Centre	Pahang (Pekan)	3°29′32″N (3.492092)	51
	Selangor	2°50′34.7″N (2.842971)	98
South	Negeri Sembilan	2°33′12.75″N (2.553541)	9
	Melaka	2°12′11.81″N (2.203281)	5
	Johor	1°27′41.98″N (1.461662)	4
	Singapore (Malay)	1°17′13.35″N (1.287043)	89

*Latitude coordinate from Yandex (http://map.yandex.com)

### Data assemblage

Data QC and assemblage were carried out with PLINK 1.07. Datasets from each platform were first filtered for individuals with >10% missing rate, > 10% SNP missing rate, minor allele frequencies (MAF) < 0.05, and Hardy-Weinberg Equilibrium (HWE) P < 0.002. Then the filtered datasets were subsequently merged, consisting 42,633 SNPs shared among all the 402 Malay samples. The dataset was further pruned down by removing any SNP with r^2^>0.8, leaving a total SNP of 41,400 for further analyses.

### Analysis of population structure

Principal Component Analysis (PCA) was first carried out using the *smartPCA* in EIGENSOFT (ver 4.0) package. The genetic component of the Malay populations was inferred with ADMIXTURE ver 1.22 (Alexander et al., 2009) [19], with the 41,400 SNPs overlapped across all samples.

### Latitude-PC correlation

Pearson’s correlation coefficient was calculated to evaluate the relationship between the genetic coordinates (PC values) and the geographic latitudes.

### Pairwise F_ST_

Unbiased estimation of F_ST_ was calculated according to Weir and Hill (2002) [20], with confidence intervals estimated by bootstrapping with 1,000 replications.

### GWAS simulation

Simulations on genome-wide association study (GWAS) were performed using PLINK 1.07, following the procedure of Xu et al. (2009) [12].

## Additional files

Additional file 1: Figure S1. PCA plot including samples from the 11 states of Peninsular Malaysia. **Figure S2.** Correlation between PC1 and longitude. Figure S3. Correlation between geographical distance and F_ST_. Additional file 2: Table S1. Regional pair-wise F_ST_ of the populations collected from different states in Malaysia. **Table S2.** Top 1% SNPs that are highly differentiated between the Malays from northern and southern region of Peninsular. **Table S3.** Gene ontology and enrichment analysis of the candidate genes underlying the top 0.1% highly differentiated SNPs between the Malays from the north and south Peninsular Malaysia. **Table S4.** Simulation analysis for GWAS before and after removing 1,666 SNPs with different minor allele present between the north and south Malays. Additional file 3:
**SNPs with different minor allele between northern and southern Malays.**
Additional file 4:
**GWAS annotation.**

## Abbreviations

GWAS: Genome wide association
FELDA: Federal land development authority
SNP: Single nucleotide polymorphism
AIMs: Ancestry informative markers
HDL-c: High density lipoprotein cholesterol
HDL-c: High density lipoprotein cholesterol
HapMap 3: Hapmap project 3
Punadj: Unadjusted P value
GC: Genomic control
Bonf: Bonfferoni correction
FDR: False discovery rate
