# Supplementary figures and images for: Fine-scale population structure of Malays in Peninsular Malaysia and Singapore and implications for association studies
Source: Hum Genomics. 2015 Jul 22;9(1):16. doi: 10.1186/s40246-015-0039-x (PMC4509480; doi:10.1186/s40246-015-0039-x)

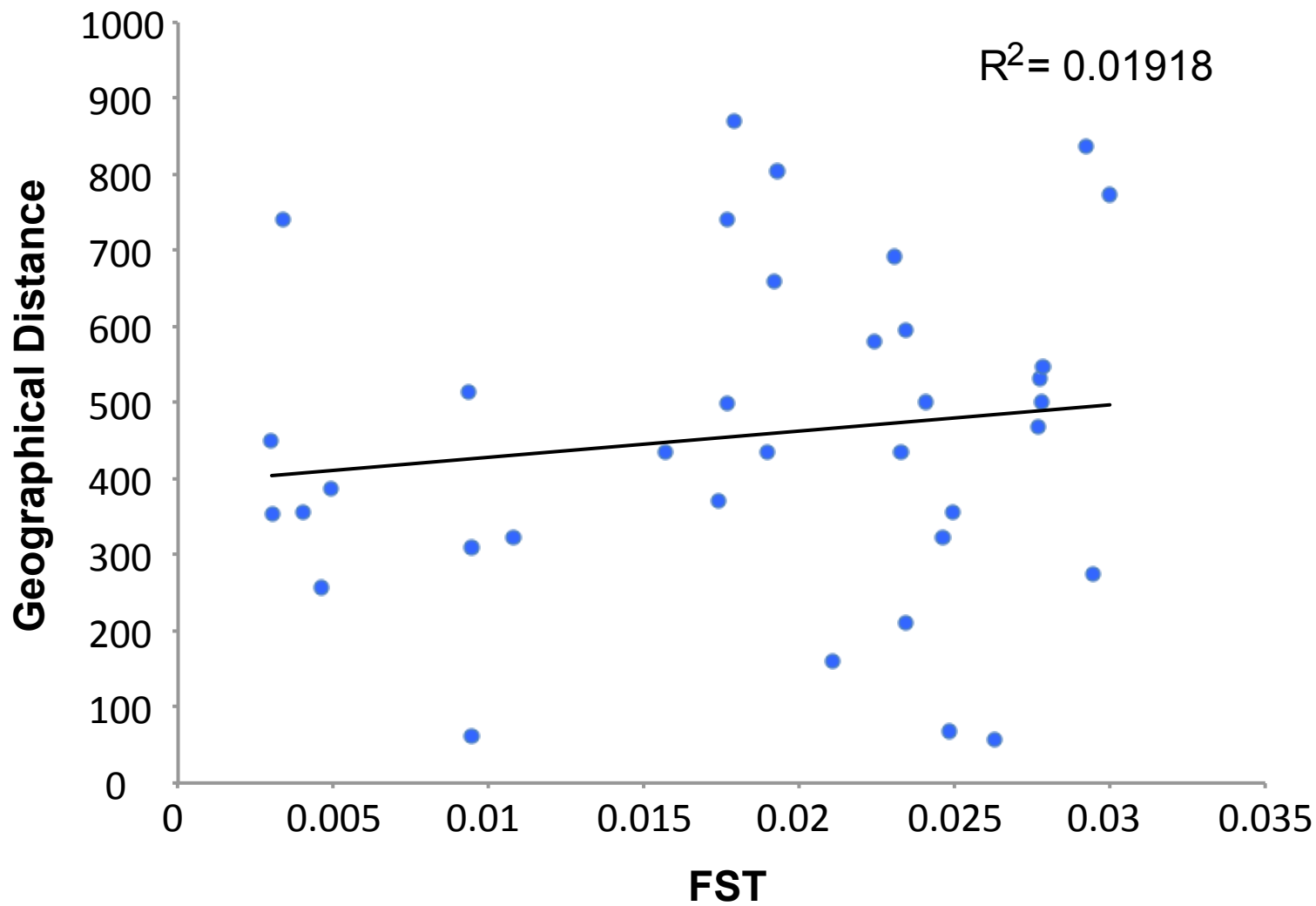

Supplement: Additional file 1: Figure S1. — PCA plot including samples from the 11 states of Peninsular Malaysia. Figure S2. Correlation between PC1 and longitude. Figure S3. Correlation between geographical distance and FST. [file 40246_2015_39_MOESM1_ESM.zip › FigureS3.pdf]

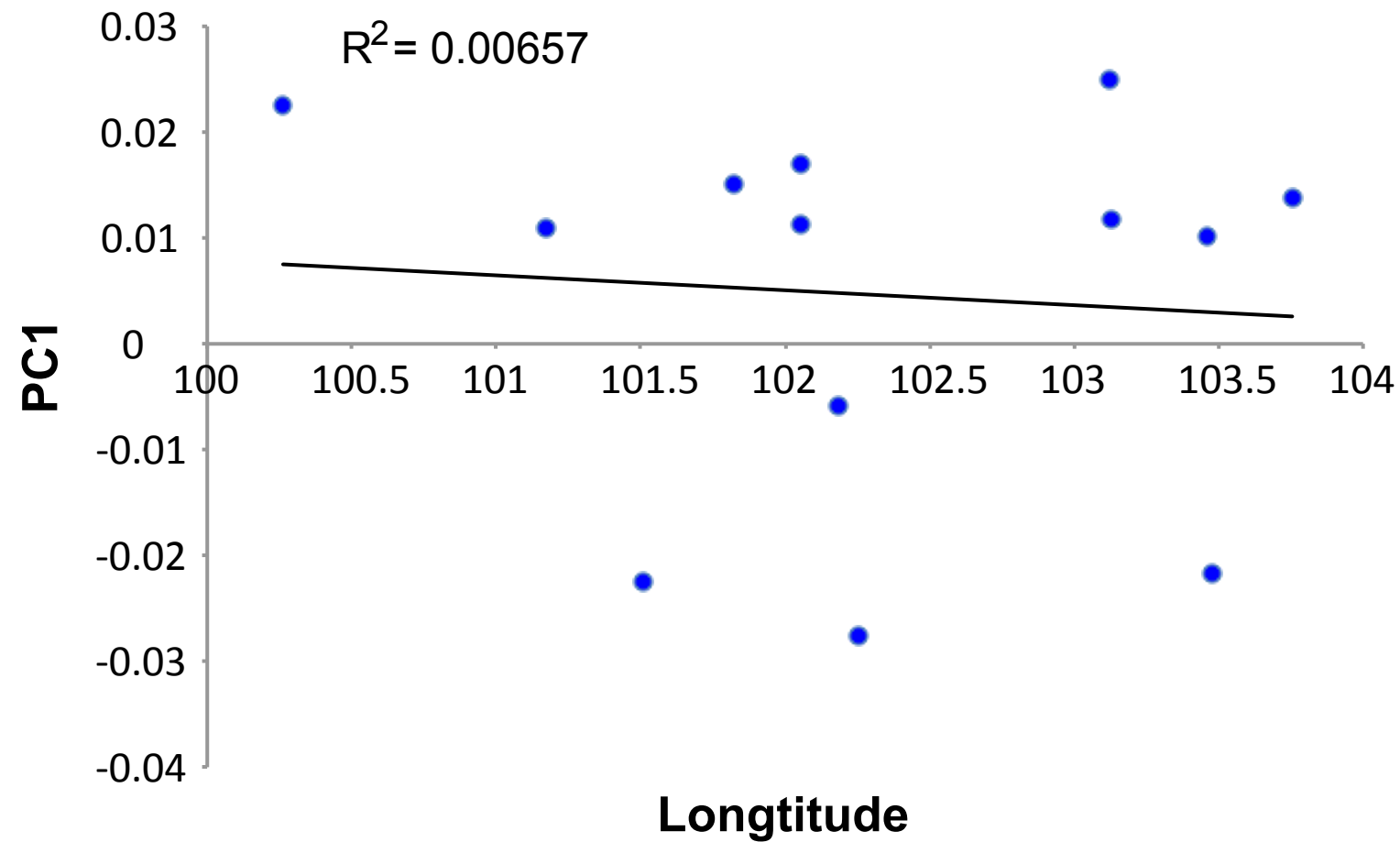

Supplement: Additional file 1: Figure S1. — PCA plot including samples from the 11 states of Peninsular Malaysia. Figure S2. Correlation between PC1 and longitude. Figure S3. Correlation between geographical distance and FST. [file 40246_2015_39_MOESM1_ESM.zip › FigureS2.pdf]

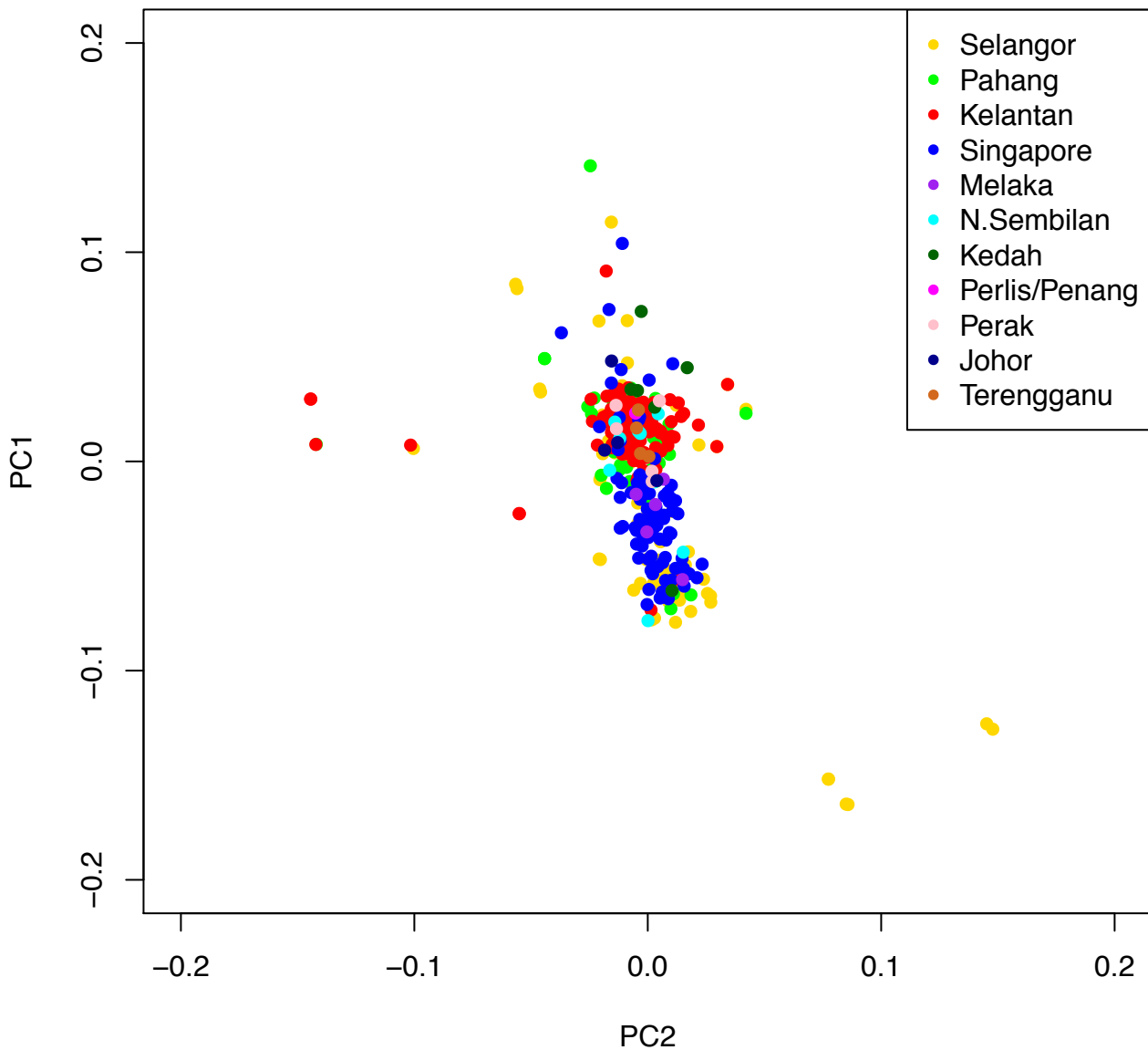

Supplement: Additional file 1: Figure S1. — PCA plot including samples from the 11 states of Peninsular Malaysia. Figure S2. Correlation between PC1 and longitude. Figure S3. Correlation between geographical distance and FST. [file 40246_2015_39_MOESM1_ESM.zip › FigureS1.pdf]
