# Supplementary material for: Fine-scale population structure of Malays in Peninsular Malaysia and Singapore and implications for association studies
Source: Hum Genomics. 2015 Jul 22;9(1):16. doi: 10.1186/s40246-015-0039-x (PMC4509480; doi:10.1186/s40246-015-0039-x)
Supplement: Additional file 2: Table S1. — Regional pair-wise FST of the populations collected from different states in Malaysia. Table S2. Top 1% SNPs that are highly differentiated between the Malays from northern and southern region of Peninsular. Table S3. Gene ontology and enrichment analysis of the candidate genes underlying the top 0.1% highly differentiated SNPs between the Malays from the north and south Peninsular Malaysia. Table S4. Simulation analysis for GWAS before and after removing 1,666 SNPs with different minor allele present between the north and south Malays. [file 40246_2015_39_MOESM2_ESM.docx]

## SUPPLEMENTARY TABLE:

**Table S1.** Regional pair-wise F_ST_

|  | **Kelantan** | **MAS** | **Kedah** | **Perlis/ Pulau Pinang /Perak** | **Pahang** | **Terengganu** | **Selangor** | **Melaka/ Negeri Sembilan** | **Johor** |
| --- | --- | --- | --- | --- | --- | --- | --- | --- | --- |
| **Kelantan** | - | 0.0033 | 0.0174 | 0.0157 | 0.0040 | 0.0211 | 0.0030 | 0.0093 | 0.0231 |
| **MAS** |  | - | 0.0193 | 0.0179 | 0.0049 | 0.0234 | 0.0031 | 0.0095 | 0.0248 |
| **Kedah** |  |  | - | 0.0263 | 0.0192 | 0.0277 | 0.0190 | 0.0241 | 0.0300 |
| **Perlis/Pulau Pinang/Perak** |  |  |  | - | 0.0177 | 0.0277 | 0.0177 | 0.0224 | 0.0292 |
| **Pahang** |  |  |  |  | - | 0.0234 | 0.0046 | 0.0108 | 0.0250 |
| **Terengganu** |  |  |  |  |  | - | 0.0233 | 0.0278 | 0.0279 |
| **Selangor** |  |  |  |  |  |  | - | 0.0094 | 0.0246 |
| **Melaka/Negeri Sembilan** |  |  |  |  |  |  |  | - | 0.0295 |
| **Johor** |  |  |  |  |  |  |  |  | - |

**Table S2.** Top 1% SNPs that are highly differentiated between the Malays from northern and southern region of Peninsular

| **rsID** | **Chr** | **Position** | **Alt allele** | **F_ST_** | **MAF_North** | **MAF_South** | **Gene** | **Category** |
| --- | --- | --- | --- | --- | --- | --- | --- | --- |
| rs4149264 | 9 | 107,677,211 | C | 0.22556278 | 0.4856 | 0.1682 | *ABCA1* | intronic |
| rs10102377 | 8 | 83,762,822 | T | 0.22505985 | 0.4097 | 0.2336 |  |  |
| rs4148475 | 13 | 95,853,574 | A | 0.22420777 | 0.4676 | 0.184 | *ABCC4* | intronic |
| rs1056836 | 2 | 38,298,203 | G | 0.20371082 | 0.4757 | 0.1934 | *CYP1B1* | coding |
| rs1126965 | 17 | 70,642,790 | G | 0.19307204 | 0.5 | 0.1822 | *SLC39A11* | 3utr |
| rs17769090 | 15 | 70,630,120 | A | 0.1635732 | 0.4648 | 0.2381 |  |  |
| rs6974363 | 7 | 47,633,187 | G | 0.14209714 | 0.493 | 0.2333 |  |  |
| rs837395 | 1 | 47,269,338 | A | 0.14000445 | 0.4897 | 0.2383 | *CYP4B1* | intronic |
| rs4646430 | 2 | 38,306,415 | G | 0.13838369 | 0.4621 | 0.1981 |  |  |
| rs215101 | 16 | 16,052,973 | G | 0.12062875 | 0.4752 | 0.271 | *ABCC1* | intronic |
| rs12920607 | 16 | 73,728,620 | C | 0.1183055 | 0.475 | 0.2736 |  |  |
| rs837398 | 1 | 47,266,422 | A | 0.11243902 | 0.4897 | 0.2664 | *CYP4B1* | intronic |
| rs809367 | 10 | 89,741,806 | A | 0.10883593 | 0.4307 | 0.2009 |  |  |
| rs316133 | 6 | 52,847,551 | C | 0.09565698 | 0.4823 | 0.2594 | *GSTA4* | intronic |
| rs6130511 | 20 | 42,681,088 | A | 0.091551 | 0.2801 | 0.1 | *TOX2* | intronic |
| rs2132845 | 4 | 140,587,125 | T | 0.09103718 | 0.4255 | 0.215 | *MGST2* | 5utr |
| rs5761313 | 22 | 26,313,745 | T | 0.08870773 | 0.4964 | 0.2804 | *MYO18B* | intronic |
| rs10485805 | 20 | 54,945,783 | G | 0.08525188 | 0.4397 | 0.2336 | *AURKA* | intronic |
| rs10489142 | 1 | 7,363,310 | G | 0.0834509 | 0.4507 | 0.3364 | *CAMTA1* | intronic |
| rs2274928 | 13 | 24,044,546 | A | 0.07789116 | 0.3601 | 0.4346 | *LINC00327* | intronic |
| rs11935505 | 4 | 145,226,422 | A | 0.07584146 | 0.04643 | 0.1682 |  |  |
| rs1566869 | 12 | 52,266,348 | A | 0.06950685 | 0.3406 | 0.1682 |  |  |
| rs1884897 | 20 | 6,612,832 | G | 0.06945538 | 0.2812 | 0.1215 |  |  |
| rs4530975 | 7 | 104,415,415 | T | 0.06789587 | 0.1862 | 0.05607 | *LHFPL3* | intronic |
| rs6024831 | 20 | 54,938,464 | G | 0.06751893 | 0.4161 | 0.3915 | *FAM210B* | intronic |
| rs1160798 | 6 | 112,438,446 | C | 0.0661181 | 0.06897 | 0.1934 | *LAMA4* | intronic |
| rs2158196 | 4 | 114,416,596 | C | 0.06575608 | 0.2391 | 0.09434 | *CAMK2D* | intronic |
| rs16961766 | 13 | 103,899,499 | A | 0.06450993 | 0.3143 | 0.1524 |  |  |
| rs10962015 | 9 | 15,387,949 | A | 0.06383759 | 0.2482 | 0.1028 |  |  |
| rs6884962 | 5 | 172,682,382 | A | 0.06233731 | 0.4896 | 0.3271 |  |  |
| rs17126776 | 12 | 39,311,625 | A | 0.06026741 | 0.1759 | 0.3318 |  |  |
| rs2755209 | 13 | 41,137,804 | C | 0.0602086 | 0.25 | 0.1075 | *FOXO1* | intronic |
| rs9783586 | 13 | 108,361,559 | T | 0.06006614 | 0.2671 | 0.4393 | *FAM155A* | intronic |
| rs10968093 | 9 | 27,753,227 | A | 0.05936945 | 0.06338 | 0.1776 |  |  |
| rs2458286 | 8 | 103,978,699 | T | 0.05905707 | 0.4424 | 0.3774 |  |  |
| rs4875364 | 8 | 4,444,592 | C | 0.05828692 | 0.3094 | 0.1557 | *CSMD1* | intronic |
| rs11145506 | 9 | 80,264,584 | T | 0.05775429 | 0.3821 | 0.217 | *GNA14* | intronic |
| rs11604366 | 11 | 28,887,766 | C | 0.05769589 | 0.2695 | 0.4387 |  |  |
| rs9881633 | 3 | 112,881,539 | T | 0.05741804 | 0.3 | 0.472 | *RP11_572M11.3* | intronic |
| rs5762448 | 22 | 28,408,444 | C | 0.05728724 | 0.3514 | 0.1916 | *TTC28* | intronic |
| rs6467991 | 7 | 83,954,737 | C | 0.05698309 | 0.344 | 0.4811 |  |  |
| rs10089677 | 8 | 122,660,248 | A | 0.05687061 | 0.2329 | 0.09813 |  |  |
| rs1923254 | 13 | 41,084,241 | G | 0.05670524 | 0.3776 | 0.2143 |  |  |
| rs7813806 | 8 | 5,142,665 | C | 0.05669963 | 0.2817 | 0.1355 |  |  |
| rs7625411 | 3 | 112,811,428 | A | 0.05644415 | 0.3403 | 0.486 |  |  |
| rs2922249 | 6 | 127,954,614 | C | 0.05641386 | 0.09441 | 0.2196 |  |  |
| rs2294088 | 8 | 124,526,607 | A | 0.05641268 | 0.4514 | 0.2804 | *FBXO32* | intronic |
| rs12289262 | 11 | 12,894,758 | T | 0.05603538 | 0.3986 | 0.2336 | *TEAD1* | intronic |
| rs4937523 | 11 | 130,347,190 | T | 0.05575465 | 0.2937 | 0.4626 | *ADAMTS15* | intronic |
| rs10807768 | 7 | 13,662,014 | A | 0.05539112 | 0.3169 | 0.1651 |  |  |
| rs976272 | 14 | 61,449,328 | A | 0.05512231 | 0.4897 | 0.3178 | *SLC38A6* | coding |
| rs13027801 | 2 | 143,602,503 | C | 0.05511875 | 0.2862 | 0.4533 |  |  |
| rs17701834 | 19 | 22,121,458 | G | 0.05491233 | 0.2172 | 0.08879 |  |  |
| rs7193843 | 16 | 54,677,292 | G | 0.05481337 | 0.1448 | 0.285 |  |  |
| rs7097885 | 10 | 16,506,501 | C | 0.05417097 | 0.2832 | 0.4486 | *PTER* | intronic |
| rs2791398 | 1 | 245,965,551 | G | 0.05403795 | 0.05944 | 0.1651 | *SMYD3* | intronic |
| rs10486802 | 7 | 39,723,768 | A | 0.05312658 | 0.1884 | 0.07009 | *RALA* | intronic |
| rs8031676 | 15 | 96,910,440 | C | 0.05292304 | 0.4306 | 0.2664 |  |  |
| rs7186479 | 16 | 82,602,736 | C | 0.05266879 | 0.2517 | 0.1168 |  |  |
| rs6054383 | 20 | 6,584,604 | T | 0.05255925 | 0.3986 | 0.2383 |  |  |
| rs4460308 | 7 | 104,420,060 | C | 0.05206173 | 0.1866 | 0.07009 | *LHFPL3* | intronic |
| rs3775779 | 4 | 70,709,207 | A | 0.05204798 | 0.476 | 0.3551 | *SULT1E1* | intronic |
| rs9375877 | 6 | 132,690,239 | G | 0.05173322 | 0.4862 | 0.3458 | *MOXD1* | intronic |
| rs2180691 | 20 | 54,964,361 | A | 0.05172836 | 0.45 | 0.2857 | *AURKA* | intronic |
| rs7778955 | 7 | 39,740,487 | G | 0.05169051 | 0.1438 | 0.04206 | *RALA* | intronic |
| rs4608114 | 12 | 92,384,658 | A | 0.05168749 | 0.4366 | 0.2736 | *C12orf79* | intronic |
| rs6946733 | 7 | 106,670,288 | A | 0.05138385 | 0.4281 | 0.2664 |  |  |
| rs816650 | 10 | 601,089 | T | 0.05136738 | 0.114 | 0.2406 | *DIP2C* | intronic |
| rs6490805 | 13 | 24,084,809 | C | 0.05072431 | 0.08394 | 0.1981 |  |  |
| rs17171480 | 7 | 35,585,669 | A | 0.05055598 | 0.09286 | 0.2103 |  |  |
| rs17015112 | 3 | 77,319,487 | G | 0.05047116 | 0.4545 | 0.3785 | *ROBO2* | intronic |
| rs573186 | 3 | 124,178,276 | C | 0.05025945 | 0.2483 | 0.1168 | *KALRN* | intronic |
| rs10752609 | 1 | 154,791,128 | A | 0.05009748 | 0.3147 | 0.1698 | *KCNN3* | intronic |
| rs1862737 | 16 | 75,281,964 | C | 0.04998496 | 0.4155 | 0.257 | *BCAR1* | 5utr |
| rs11941589 | 4 | 140,115,744 | A | 0.0498735 | 0.3904 | 0.4439 |  |  |
| rs560096 | 11 | 68,678,962 | C | 0.04973148 | 0.4653 | 0.3692 | *IGHMBP2* | coding |
| rs730278 | 1 | 172,603,940 | T | 0.04966739 | 0.3697 | 0.217 |  |  |
| rs6861345 | 5 | 129,552,816 | G | 0.04941344 | 0.3732 | 0.4623 |  |  |
| rs10789515 | 1 | 48,212,615 | C | 0.04902148 | 0.1621 | 0.05607 |  |  |
| rs2301871 | 7 | 43,298,065 | A | 0.04874052 | 0.1099 | 0.2311 | *HECW1* | intronic |
| rs9319027 | 13 | 84,763,926 | A | 0.04815165 | 0.1879 | 0.07477 | *LINC00333* | intronic |
| rs10898250 | 11 | 84,222,437 | G | 0.04802421 | 0.2808 | 0.1449 | *DLG2* | intronic |
| rs4340119 | 12 | 61,613,734 | T | 0.04800626 | 0.4583 | 0.2991 |  |  |
| rs10510248 | 3 | 3,048,519 | C | 0.0479066 | 0.1931 | 0.3349 | *CNTN4* | intronic |
| rs7119876 | 11 | 17,537,033 | G | 0.04729868 | 0.1667 | 0.06075 | *USH1C* | intronic |
| rs6918777 | 6 | 93,369,655 | T | 0.04723717 | 0.4317 | 0.2757 |  |  |
| rs11074359 | 16 | 19,085,298 | T | 0.04720456 | 0.2 | 0.08411 | *COQ7* | coding |
| rs11746690 | 5 | 149,143,963 | A | 0.04715634 | 0.05556 | 0.1509 | *PPARGC1B* | intronic |
| rs4811693 | 20 | 54,937,543 | T | 0.04711564 | 0.4231 | 0.4151 | *FAM210B* | intronic |
| rs12550829 | 8 | 8,506,404 | C | 0.04708823 | 0.4965 | 0.3364 |  |  |
| rs2888611 | 7 | 87,103,670 | G | 0.04691879 | 0.4414 | 0.285 | *ABCB4* | intronic |
| rs2243057 | 5 | 76,127,135 | A | 0.04666593 | 0.3733 | 0.2243 | *F2RL1* | intronic |
| rs885704 | 19 | 2,833,431 | G | 0.04664306 | 0.4896 | 0.3505 | *ZNF554* | intronic |
| rs10506294 | 12 | 51,070,554 | C | 0.04656495 | 0.4897 | 0.3505 | *DIP2B* | intronic |
| rs12294573 | 11 | 24,087,714 | T | 0.04648634 | 0.4857 | 0.3271 |  |  |
| rs7786816 | 7 | 155,312,325 | C | 0.04634304 | 0.3333 | 0.4907 | *CNPY1* | intronic |
| rs856215 | 14 | 59,240,290 | C | 0.04624432 | 0.2837 | 0.1495 |  |  |
| rs10503343 | 8 | 5,793,209 | A | 0.04594985 | 0.4897 | 0.3318 |  |  |
| rs7785709 | 7 | 7,938,344 | A | 0.04590533 | 0.4897 | 0.3318 |  |  |
| rs524373 | 11 | 30,027,074 | G | 0.04557641 | 0.1893 | 0.3271 |  |  |
| rs17194904 | 6 | 132,899,017 | T | 0.04546749 | 0.2289 | 0.1075 |  |  |
| rs7490744 | 13 | 84,976,969 | G | 0.04536931 | 0.4966 | 0.3458 | *LINC00333* | intronic |
| rs2722276 | 7 | 37,989,178 | A | 0.04512089 | 0.3662 | 0.4766 | *EPDR1* | intronic |
| rs7802120 | 7 | 19,758,362 | G | 0.04505376 | 0.4281 | 0.2757 | *TMEM196* | intronic |
| rs2071519 | 8 | 120,435,908 | G | 0.04501862 | 0.2345 | 0.1121 | *NOV* | 3utr |
| rs17698191 | 7 | 35,548,431 | G | 0.04501657 | 0.0931 | 0.2028 |  |  |
| rs9863587 | 3 | 197,581,147 | G | 0.04491578 | 0.3356 | 0.4907 | *LRCH3* | intronic |
| rs272531 | 1 | 44,988,433 | A | 0.04479371 | 0.1644 | 0.06132 | *RNF220* | intronic |
| rs16838771 | 1 | 157,511,228 | C | 0.04476225 | 0.06897 | 0.1682 | *FCRL5* | intronic |
| rs17112735 | 1 | 56,207,532 | C | 0.04462196 | 0.3714 | 0.472 |  |  |
| rs2942133 | 1 | 204,369,875 | G | 0.04458759 | 0.493 | 0.3505 |  |  |
| rs1981035 | 9 | 112,663,125 | T | 0.04453111 | 0.161 | 0.2905 | *PALM2* | intronic |
| rs11695317 | 2 | 38,687,498 | C | 0.04429964 | 0.3901 | 0.4533 | *AC016995.3* | intronic |
| rs10949240 | 6 | 14,353,238 | A | 0.04415042 | 0.2806 | 0.4299 |  |  |
| rs7229380 | 18 | 2,094,574 | A | 0.04407169 | 0.2143 | 0.09813 |  |  |
| rs7929114 | 11 | 45,706,162 | G | 0.04393155 | 0.4276 | 0.4159 |  |  |
| rs1997021 | 18 | 27,676,662 | A | 0.04390547 | 0.2568 | 0.1308 |  |  |
| rs6563698 | 13 | 39,934,077 | A | 0.0437882 | 0.3517 | 0.2095 | *LHFP* | intronic |
| rs6548344 | 3 | 164,076,198 | A | 0.04340865 | 0.2734 | 0.1449 |  |  |
| rs6741957 | 2 | 57,077,745 | A | 0.04336729 | 0.2676 | 0.1402 |  |  |
| rs16906530 | 8 | 80,046,610 | C | 0.04333178 | 0.4379 | 0.4065 |  |  |
| rs13273033 | 8 | 9,540,693 | A | 0.04332559 | 0.4854 | 0.3598 | *TNKS* | intronic |
| rs6899876 | 6 | 41,796,970 | C | 0.04330503 | 0.2569 | 0.4019 | *USP49* | intronic |
| rs2015461 | 15 | 35,119,748 | T | 0.04277639 | 0.3973 | 0.4486 |  |  |
| rs940199 | 11 | 21,069,877 | A | 0.04277156 | 0.4483 | 0.3972 | *NELL1* | intronic |
| rs12577901 | 11 | 125,123,425 | C | 0.04248039 | 0.3979 | 0.2523 | *PKNOX2* | intronic |
| rs12455952 | 18 | 58,840,518 | G | 0.04241794 | 0.1062 | 0.217 |  |  |
| rs951629 | 2 | 9,796,791 | A | 0.0424086 | 0.4184 | 0.271 |  |  |
| rs2022448 | 6 | 26,207,928 | A | 0.04205161 | 0.1727 | 0.07009 |  |  |
| rs250744 | 5 | 76,041,910 | A | 0.04199226 | 0.3759 | 0.472 |  |  |
| rs732380 | 3 | 60,276,722 | T | 0.04187865 | 0.3785 | 0.2358 | *FHIT* | intronic |
| rs42398 | 5 | 96,120,455 | T | 0.04163457 | 0.3767 | 0.4717 | *ERAP1* | intronic |
| rs4578488 | 12 | 13,424,929 | T | 0.04154614 | 0.3188 | 0.4673 |  |  |
| rs11167118 | 8 | 143,108,079 | T | 0.04149141 | 0.3617 | 0.2217 |  |  |
| rs10876099 | 12 | 51,231,061 | C | 0.04138163 | 0.3453 | 0.4952 |  |  |
| rs7732591 | 5 | 142,125,177 | C | 0.04137284 | 0.3151 | 0.4626 | *AC005592.1* | non-coding |
| rs12234107 | 5 | 67,221,604 | G | 0.04136469 | 0.1199 | 0.2336 |  |  |
| rs9394699 | 6 | 40,449,290 | A | 0.04122382 | 0.1644 | 0.2897 | *LRFN2* | intronic |
| rs10491196 | 17 | 54,215,810 | C | 0.04119152 | 0.1759 | 0.3037 |  |  |
| rs4659986 | 1 | 240,799,543 | C | 0.04117327 | 0.2393 | 0.3785 |  |  |
| rs4576485 | 9 | 113,815,164 | A | 0.04089855 | 0.1642 | 0.06542 |  |  |
| rs4722596 | 7 | 26,491,234 | T | 0.04081125 | 0.09589 | 0.2009 | *LOC441204* | intronic |
| rs2027626 | 3 | 37,662,867 | G | 0.04063123 | 0.2568 | 0.3972 | *ITGA9* | intronic |
| rs7829575 | 8 | 136,444,089 | T | 0.04057815 | 0.07554 | 0.1729 |  |  |
| rs731727 | 11 | 34,857,834 | G | 0.04055739 | 0.1507 | 0.05607 |  |  |
| rs7157202 | 14 | 59,875,592 | T | 0.04045441 | 0.2935 | 0.1651 |  |  |
| rs12220359 | 10 | 5,877,213 | G | 0.04037075 | 0.05986 | 0.1495 |  |  |
| rs12784400 | 10 | 119,611,204 | A | 0.04027862 | 0.2089 | 0.09813 |  |  |
| rs7765655 | 6 | 132,897,858 | A | 0.04024073 | 0.2911 | 0.4346 |  |  |
| rs980937 | 1 | 158,140,205 | A | 0.04023784 | 0.1503 | 0.271 |  |  |
| rs11030551 | 11 | 29,044,894 | C | 0.04009463 | 0.2945 | 0.4381 |  |  |
| rs6763039 | 3 | 11,096,975 | T | 0.04008762 | 0.3462 | 0.2103 |  |  |
| rs11001968 | 10 | 78,772,456 | G | 0.03999996 | 0.2329 | 0.1168 | *KCNMA1* | intronic |
| rs11099756 | 4 | 151,063,917 | G | 0.0399679 | 0.2361 | 0.3726 | *DCLK2* | intronic |
| rs10483324 | 14 | 27,293,442 | C | 0.03993947 | 0.1931 | 0.3224 |  |  |
| rs1453998 | 11 | 21,183,993 | C | 0.03989675 | 0.4645 | 0.3178 | *NELL1* | intronic |
| rs11112414 | 12 | 105,613,957 | A | 0.03988145 | 0.4345 | 0.4159 | *APPL2* | intronic |
| rs2065841 | 1 | 61,728,597 | G | 0.03982411 | 0.3438 | 0.4907 | *NFIA* | intronic |
| rs1864207 | 19 | 42,428,767 | G | 0.03961703 | 0.2414 | 0.1238 |  |  |
| rs4806877 | 19 | 2,846,557 | G | 0.03912701 | 0.4829 | 0.3364 | *ZNF555* | intronic |
| rs739231 | 22 | 44,282,276 | G | 0.03891912 | 0.1552 | 0.06075 | *PNPLA5* | coding |
| rs705648 | 2 | 216,933,792 | T | 0.03874868 | 0.2432 | 0.3785 | *PECR* | intronic |
| rs7198109 | 16 | 18,074,463 | G | 0.03874762 | 0.4041 | 0.4486 |  |  |
| rs17138455 | 7 | 17,965,838 | A | 0.03873898 | 0.266 | 0.1449 | *SNX13* | intronic |
| rs10849774 | 12 | 121,082,228 | T | 0.03866652 | 0.2257 | 0.3585 | *CABP1* | intronic |
| rs10894646 | 11 | 132,935,388 | C | 0.0384929 | 0.2123 | 0.1028 | *OPCML* | intronic |
| rs7011968 | 8 | 21,308,489 | T | 0.03844966 | 0.2218 | 0.3538 |  |  |
| rs7109883 | 11 | 134,369,417 | T | 0.03839582 | 0.3486 | 0.215 | *LOC283177* | intronic |
| rs4950949 | 1 | 202,810,562 | T | 0.03833133 | 0.3821 | 0.4717 |  |  |
| rs12898022 | 14 | 103,351,529 | T | 0.03831387 | 0.2179 | 0.3491 | *TRAF3* | intronic |
| rs11857366 | 15 | 99,263,801 | G | 0.03826629 | 0.4897 | 0.3443 | *IGF1R* | intronic |
| rs12507764 | 4 | 70,756,086 | A | 0.03825594 | 0.3321 | 0.2009 |  |  |
| rs4713206 | 6 | 11,146,875 | A | 0.03815033 | 0.3333 | 0.4766 |  |  |
| rs1756339 | 14 | 43,433,535 | A | 0.038048 | 0.2226 | 0.3538 |  |  |
| rs8047615 | 16 | 82,634,053 | G | 0.03804312 | 0.2055 | 0.09813 |  |  |
| rs11233872 | 11 | 83,709,994 | A | 0.03803791 | 0.06849 | 0.1589 | *DLG2* | intronic |
| rs3733876 | 5 | 176,382,995 | A | 0.03795228 | 0.1724 | 0.2944 | *UIMC1* | coding |
| rs10208925 | 2 | 79,152,829 | C | 0.03777121 | 0.5 | 0.3551 |  |  |
| rs7000337 | 8 | 14,203,326 | T | 0.03775387 | 0.3472 | 0.4907 | *SGCZ* | intronic |
| rs4148366 | 16 | 16,195,565 | G | 0.03771073 | 0.338 | 0.481 | *ABCC1* | intronic |
| rs11746728 | 5 | 174,050,806 | C | 0.03770403 | 0.2695 | 0.4065 |  |  |
| rs880090 | 19 | 19,740,729 | G | 0.03762028 | 0.4555 | 0.3131 | *GMIP* | 3utr |
| rs6518752 | 22 | 31,999,127 | A | 0.03760309 | 0.2183 | 0.1085 | *SFI1* | intronic |
| rs10824587 | 10 | 79,740,262 | C | 0.0374593 | 0.1793 | 0.3019 | *POLR3A* | intronic |
| rs6727485 | 2 | 120,130,796 | T | 0.03741336 | 0.1207 | 0.229 | *DBI* | intronic |
| rs3124236 | 9 | 90,211,490 | A | 0.03740898 | 0.339 | 0.4813 | *DAPK1* | intronic |
| rs10514031 | 18 | 69,663,285 | T | 0.03733446 | 0.45 | 0.3084 |  |  |
| rs1001408 | 12 | 67,292,288 | C | 0.03725866 | 0.07241 | 0.1636 |  |  |
| rs4775287 | 15 | 60,987,230 | T | 0.03713078 | 0.2363 | 0.3679 | *RORA* | intronic |
| rs4731190 | 7 | 79,701,003 | C | 0.03712794 | 0.4161 | 0.4393 |  |  |
| rs7867860 | 9 | 108,759,480 | T | 0.03709989 | 0.1507 | 0.2664 |  |  |
| rs1497482 | 4 | 55,797,131 | A | 0.0370542 | 0.4929 | 0.3632 |  |  |
| rs1399956 | 2 | 172,510,689 | G | 0.03704791 | 0.2517 | 0.1355 |  |  |
| rs11739747 | 5 | 101,397,849 | A | 0.03687767 | 0.1849 | 0.08411 |  |  |
| rs12674179 | 7 | 43,212,606 | T | 0.03674314 | 0.2586 | 0.3925 | *HECW1* | intronic |
| rs834485 | 1 | 64,484,485 | T | 0.03672756 | 0.3241 | 0.1963 | *ROR1* | intronic |
| rs17060428 | 4 | 175,167,253 | A | 0.03670014 | 0.3759 | 0.4813 | *FBXO8* | intronic |
| rs1868090 | 2 | 46,638,563 | G | 0.03667811 | 0.3309 | 0.4717 |  |  |
| rs10053925 | 5 | 78,220,029 | C | 0.03666332 | 0.07292 | 0.1636 | *ARSB* | intronic |
| rs2235674 | 6 | 155,771,008 | T | 0.0366343 | 0.4792 | 0.3774 | *NOX3* | intronic |
| rs4706352 | 6 | 90,609,718 | C | 0.03661615 | 0.4965 | 0.3538 |  |  |
| rs12644119 | 4 | 90,603,419 | A | 0.03661513 | 0.3669 | 0.4906 |  |  |
| rs7975586 | 12 | 63,666,478 | T | 0.03653969 | 0.2273 | 0.1168 |  |  |
| rs12624916 | 20 | 1,295,639 | A | 0.03640815 | 0.4927 | 0.3505 | *SDCBP2* | intronic |
| rs8043993 | 16 | 19,049,378 | G | 0.03636253 | 0.2163 | 0.1085 | *TMC7* | intronic |
| rs2144825 | 14 | 103,290,938 | T | 0.03632617 | 0.1747 | 0.2944 | *TRAF3* | intronic |
| rs10152333 | 15 | 86,854,359 | C | 0.03632219 | 0.4281 | 0.2897 | *AGBL1* | intronic |
| rs10949239 | 6 | 14,322,665 | C | 0.03632219 | 0.4281 | 0.2897 |  |  |
| rs1314336 | 4 | 103,378,866 | T | 0.03631001 | 0.4861 | 0.3443 |  |  |
| rs12672192 | 7 | 130,118,002 | G | 0.03626656 | 0.331 | 0.2028 |  |  |
| rs2356350 | 2 | 192,002,538 | A | 0.03616677 | 0.4552 | 0.4019 | *STAT4* | intronic |
| rs6681392 | 1 | 154,796,712 | C | 0.03615063 | 0.4041 | 0.4533 | *KCNN3* | intronic |
| rs4816257 | 21 | 26,977,006 | G | 0.03613375 | 0.09028 | 0.1869 | *MRPL39* | intronic |
| rs1014448 | 12 | 12,746,585 | T | 0.03612573 | 0.04196 | 0.1168 |  |  |
| rs6818398 | 4 | 153,943,571 | C | 0.03603079 | 0.1986 | 0.3224 |  |  |
| rs10759237 | 9 | 110,214,347 | A | 0.03600457 | 0.2143 | 0.1075 |  |  |
| rs10871427 | 16 | 82,521,530 | C | 0.03598584 | 0.3459 | 0.486 |  |  |
| rs10275875 | 7 | 144,204,965 | T | 0.0359855 | 0.339 | 0.2103 | *TPK1* | intronic |
| rs10440412 | 4 | 126,734,051 | W | 0.03581387 | 0.4144 | 0.4434 |  |  |
| rs2149642 | 20 | 7,014,445 | C | 0.03575627 | 0.3493 | 0.2196 |  |  |
| rs9809852 | 3 | 138,696,439 | C | 0.03574763 | 0.4521 | 0.3131 |  |  |
| rs10863703 | 1 | 208,375,738 | T | 0.03563799 | 0.2552 | 0.1402 | *PLXNA2* | intronic |
| rs6951643 | 7 | 126,429,517 | G | 0.03558206 | 0.2138 | 0.1075 | *GRM8* | intronic |
| rs9651453 | 10 | 106,207,209 | G | 0.03545199 | 0.1875 | 0.3084 | *CCDC147* | intronic |
| rs7335200 | 13 | 23,657,518 | A | 0.03542871 | 0.195 | 0.09346 |  |  |
| rs11125078 | 2 | 46,693,963 | G | 0.03537879 | 0.3556 | 0.4953 |  |  |
| rs11604506 | 11 | 42,649,538 | T | 0.03537789 | 0.09574 | 0.1934 |  |  |
| rs17655367 | 8 | 14,936,278 | C | 0.03536161 | 0.1644 | 0.2804 | *SGCZ* | intronic |
| rs12454179 | 18 | 2,875,255 | A | 0.03530952 | 0.2133 | 0.1075 | *EMILIN2* | intronic |
| rs10521292 | 17 | 14,898,473 | T | 0.03530247 | 0.4007 | 0.2664 |  |  |
| rs2049847 | 3 | 60,277,771 | G | 0.03529625 | 0.2429 | 0.1308 | *FHIT* | intronic |
| rs10486666 | 7 | 35,547,956 | C | 0.03528207 | 0.1918 | 0.3131 |  |  |
| rs7313392 | 12 | 115,088,212 | A | 0.03522236 | 0.4103 | 0.4486 |  |  |
| rs17779005 | 18 | 56,813,415 | A | 0.03521748 | 0.169 | 0.2857 | *SEC11C* | intronic |
| rs9366426 | 6 | 22,064,639 | C | 0.03519063 | 0.316 | 0.1916 | *CASC15* | intronic |
| rs4917328 | 8 | 142,990,575 | T | 0.03516674 | 0.2192 | 0.1121 |  |  |
| rs7834060 | 8 | 143,222,202 | A | 0.03515011 | 0.2826 | 0.1636 |  |  |
| rs10828853 | 10 | 25,997,959 | G | 0.03514596 | 0.1014 | 0.2009 | *LINC00836* | intronic |
| rs3087949 | 1 | 203,047,621 | A | 0.0350886 | 0.3103 | 0.1869 | *PPFIA4* | 3utr |
| rs4360333 | 8 | 121,686,293 | G | 0.03507125 | 0.2882 | 0.1682 | *SNTB1* | intronic |
| rs16856152 | 1 | 232,147,210 | T | 0.03505525 | 0.161 | 0.2757 | *DISC1* | intronic |
| rs7002395 | 8 | 53,605,442 | G | 0.0349377 | 0.1748 | 0.2925 | *RB1CC1* | intronic |
| rs2993561 | 13 | 96,044,531 | G | 0.03491727 | 0.4555 | 0.3178 |  |  |
| rs4658649 | 1 | 244,854,821 | A | 0.03490577 | 0.1164 | 0.2196 | *DESI2* | intronic |
| rs2755237 | 13 | 41,109,429 | C | 0.03487439 | 0.2203 | 0.3458 |  |  |
| rs2836043 | 21 | 39,272,137 | A | 0.03482018 | 0.3261 | 0.2009 | *KCNJ6* | intronic |
| rs16895458 | 5 | 65,983,977 | G | 0.03478798 | 0.2534 | 0.3832 | *MAST4* | intronic |
| rs749818 | 19 | 46,496,793 | C | 0.03478097 | 0.2708 | 0.1542 | *CCDC61* | intronic |
| rs17114382 | 1 | 57,270,093 | A | 0.03475671 | 0.1575 | 0.271 | *C1orf168* | intronic |
| rs1024782 | 19 | 46,887,567 | T | 0.03475108 | 0.3368 | 0.2103 | *PPP5C* | intronic |
| rs9480754 | 6 | 107,549,447 | G | 0.03461868 | 0.4126 | 0.2783 | *PDSS2* | intronic |
| rs9351229 | 6 | 90,629,110 | G | 0.0345735 | 0.4795 | 0.3411 |  |  |
| rs10847874 | 12 | 129,925,139 | A | 0.03451832 | 0.4301 | 0.4299 | *TMEM132D* | intronic |
| rs6748566 | 2 | 41,885,773 | A | 0.03448937 | 0.3617 | 0.5 |  |  |
| rs17160199 | 7 | 85,444,989 | C | 0.03448826 | 0.1062 | 0.2056 |  |  |
| rs9296440 | 6 | 44,472,854 | G | 0.0344772 | 0.2645 | 0.1495 |  |  |
| rs10517522 | 4 | 39,453,790 | G | 0.03443542 | 0.3664 | 0.4953 | *RPL9* | intronic |
| rs569724 | 11 | 30,023,175 | T | 0.03443474 | 0.2049 | 0.3271 |  |  |
| rs13080059 | 3 | 134,029,172 | T | 0.03441043 | 0.2759 | 0.1589 |  |  |
| rs9301668 | 13 | 90,928,965 | A | 0.03441043 | 0.2759 | 0.1589 |  |  |
| rs7223911 | 17 | 77,232,086 | T | 0.03436952 | 0.4085 | 0.4519 | *RBFOX3* | intronic |
| rs12751444 | 1 | 7,208,217 | C | 0.03427528 | 0.3143 | 0.1916 | *CAMTA1* | intronic |
| rs771374 | 11 | 10,865,739 | C | 0.03426718 | 0.4306 | 0.4299 |  |  |
| rs1863464 | 15 | 26,938,488 | A | 0.03423249 | 0.2923 | 0.1729 | *GABRB3* | intronic |
| rs3960630 | 16 | 11,270,904 | A | 0.03419129 | 0.3182 | 0.4533 | *CLEC16A* | intronic |
| rs297279 | 4 | 27,680,483 | C | 0.03417151 | 0.3357 | 0.2103 |  |  |
| rs2302312 | 17 | 56,282,763 | T | 0.0341619 | 0.15 | 0.0619 | *EPX* | intronic |
| rs10498647 | 14 | 96,381,342 | T | 0.03416083 | 0.05137 | 0.1286 | *TUNAR* | intronic |
| rs1978503 | 18 | 53,664,282 | G | 0.03412297 | 0.08562 | 0.1776 |  |  |
| rs755403 | 4 | 6,389,642 | C | 0.0340921 | 0.4932 | 0.3551 | *PPP2R2C* | intronic |
| rs1030328 | 2 | 160,386,260 | G | 0.03407152 | 0.3185 | 0.4533 | *BAZ2B* | intronic |
| rs7981942 | 13 | 80,623,346 | C | 0.03403042 | 0.25 | 0.1381 |  |  |
| rs9540413 | 13 | 65,992,984 | G | 0.03400341 | 0.1678 | 0.07477 |  |  |
| rs12868856 | 13 | 106,797,716 | T | 0.03399304 | 0.1866 | 0.08879 |  |  |
| rs9388989 | 6 | 132,697,582 | A | 0.0339135 | 0.4486 | 0.3131 | *MOXD1* | intronic |
| rs11047887 | 12 | 25,348,672 | C | 0.03390478 | 0.2637 | 0.1495 | *CASC1* | intronic |
| rs41238 | 7 | 144,186,355 | G | 0.03386708 | 0.3459 | 0.2196 | *TPK1* | intronic |
| rs7319398 | 13 | 71,439,474 | G | 0.03381801 | 0.2972 | 0.4299 |  |  |
| rs10484820 | 6 | 139,858,293 | G | 0.03375687 | 0.125 | 0.229 |  |  |
| rs9565647 | 13 | 81,890,743 | G | 0.03374566 | 0.1454 | 0.2547 |  |  |
| rs11851625 | 14 | 30,314,159 | C | 0.03373512 | 0.3322 | 0.4673 | *PRKD1* | intronic |
| rs3949904 | 1 | 62,848,090 | A | 0.03371122 | 0.3767 | 0.486 |  |  |
| rs7298165 | 12 | 32,702,740 | C | 0.03365601 | 0.1862 | 0.08879 | *FGD4* | intronic |
| rs1202047 | 20 | 59,331,325 | A | 0.03365436 | 0.4028 | 0.271 |  |  |
| rs1357365 | 17 | 34,436,532 | A | 0.03364141 | 0.3904 | 0.472 |  |  |
| rs130413 | 22 | 28,488,105 | T | 0.03361482 | 0.3077 | 0.1869 | *TTC28* | intronic |
| rs6972153 | 7 | 39,544,787 | A | 0.03361023 | 0.4078 | 0.2757 |  |  |
| rs17055518 | 3 | 55,653,812 | A | 0.03357573 | 0.3664 | 0.2383 | *ERC2* | intronic |
| rs10196846 | 2 | 38,379,110 | A | 0.03353993 | 0.1541 | 0.06542 | *CYP1B1_AS1* | intronic |
| rs11102479 | 1 | 113,006,804 | A | 0.03349888 | 0.4648 | 0.3972 |  |  |
| rs3019920 | 8 | 108,068,283 | C | 0.03349521 | 0.3681 | 0.4953 |  |  |
| rs986376 | 3 | 143,017,187 | C | 0.03347003 | 0.4075 | 0.2757 | *SLC9A9* | intronic |
| rs17016910 | 4 | 91,287,204 | A | 0.03344772 | 0.1179 | 0.2196 | *CCSER1* | intronic |
| rs273995 | 7 | 137,629,146 | T | 0.03343904 | 0.3451 | 0.2196 | *CREB3L2* | intronic |
| rs2406747 | 4 | 138,367,353 | T | 0.0334102 | 0.4286 | 0.4333 |  |  |
| rs4917653 | 10 | 115,198,443 | G | 0.03335269 | 0.4225 | 0.2897 |  |  |
| rs4670981 | 2 | 40,119,060 | G | 0.03330406 | 0.1748 | 0.2897 |  |  |
| rs4814204 | 20 | 13,110,773 | G | 0.03327846 | 0.469 | 0.3333 | *SPTLC3* | intronic |
| rs1578558 | 9 | 15,855,317 | C | 0.03327211 | 0.2396 | 0.1308 | *CCDC171* | intronic |
| rs17501615 | 3 | 84,806,803 | A | 0.03327052 | 0.2411 | 0.1321 | *LINC00971* | intronic |
| rs17067250 | 5 | 166,025,196 | G | 0.0332694 | 0.1336 | 0.0514 |  |  |
| rs9373932 | 6 | 107,564,700 | C | 0.03325764 | 0.5 | 0.3632 | *PDSS2* | intronic |
| rs2205254 | 21 | 15,725,642 | G | 0.03325398 | 0.2295 | 0.1226 |  |  |
| rs4448804 | 13 | 39,529,226 | A | 0.0332467 | 0.2158 | 0.1121 |  |  |
| rs7022345 | 9 | 7,173,752 | G | 0.03323261 | 0.2517 | 0.3785 | *KDM4C* | intronic |
| rs12491760 | 3 | 61,749,709 | G | 0.03309806 | 0.2958 | 0.1776 | *PTPRG* | intronic |
| rs6772196 | 3 | 151,147,968 | T | 0.03305163 | 0.3333 | 0.4673 | *MED12L* | intronic |
| rs12154459 | 7 | 146,254,508 | A | 0.03303546 | 0.387 | 0.4766 | *CNTNAP2* | intronic |
| rs728845 | 8 | 14,286,552 | G | 0.03300948 | 0.4896 | 0.3738 | *SGCZ* | intronic |
| rs12533012 | 7 | 70,143,677 | A | 0.03300581 | 0.4897 | 0.3538 | *AUTS2* | intronic |
| rs1956556 | 14 | 60,818,257 | G | 0.03298603 | 0.475 | 0.3396 |  |  |
| rs12580887 | 12 | 13,437,268 | C | 0.03296221 | 0.1007 | 0.1963 |  |  |
| rs4362897 | 4 | 141,304,666 | C | 0.03295017 | 0.4897 | 0.3738 | *SCOC* | intronic |
| rs16830067 | 2 | 152,331,745 | G | 0.0329337 | 0.1952 | 0.3131 | *RIF1* | 3utr |
| rs2299219 | 7 | 86,417,845 | C | 0.03284265 | 0.1937 | 0.3113 | *GRM3* | intronic |
| rs2305688 | 16 | 70,779,144 | T | 0.03279149 | 0.4101 | 0.4533 | *VAC14* | intronic |
| rs1593086 | 19 | 42,220,409 | T | 0.03279018 | 0.2234 | 0.3458 | *CEACAM5* | intronic |
| rs7227144 | 18 | 68,816,968 | C | 0.03274512 | 0.4514 | 0.3178 |  |  |
| rs135582 | 22 | 49,179,000 | T | 0.03260656 | 0.1414 | 0.2477 |  |  |
| rs1980007 | 2 | 115,756,457 | G | 0.03258883 | 0.375 | 0.2477 | *DPP10* | intronic |
| rs385674 | 21 | 46,542,670 | A | 0.03248309 | 0.2902 | 0.4198 | *ADARB1* | intronic |
| rs10879274 | 12 | 71,633,978 | T | 0.03239725 | 0.3973 | 0.4673 |  |  |
| rs11155282 | 6 | 100,593,022 | G | 0.03239389 | 0.3204 | 0.4524 |  |  |
| rs10808793 | 8 | 74,060,655 | T | 0.03234915 | 0.3562 | 0.2311 |  |  |
| rs4240286 | 4 | 114,452,724 | C | 0.03229621 | 0.3483 | 0.2243 | *CAMK2D* | intronic |
| rs11871341 | 17 | 54,335,266 | A | 0.03225918 | 0.1268 | 0.229 | *ANKFN1* | intronic |
| rs12641040 | 4 | 5,532,985 | G | 0.03223444 | 0.4486 | 0.316 |  |  |
| rs1503293 | 4 | 167,010,685 | C | 0.03215575 | 0.375 | 0.4907 | *TLL1* | intronic |
| rs7541400 | 1 | 98,846,773 | C | 0.03213368 | 0.226 | 0.1215 |  |  |
| rs6507544 | 18 | 41,339,382 | C | 0.03211949 | 0.3219 | 0.4533 |  |  |
| rs7862883 | 9 | 100,279,009 | G | 0.03211826 | 0.2329 | 0.3551 | *TMOD1* | intronic |
| rs875162 | 2 | 186,896,757 | G | 0.03211817 | 0.4452 | 0.3131 | *AC097500.2* | intronic |
| rs4771308 | 13 | 99,266,781 | A | 0.03199609 | 0.425 | 0.2944 |  |  |
| rs2265326 | 13 | 66,061,553 | T | 0.03198536 | 0.191 | 0.09434 |  |  |
| rs9363983 | 6 | 69,818,593 | T | 0.03193794 | 0.2603 | 0.1495 | *BAI3* | intronic |
| rs12325243 | 16 | 54,742,518 | T | 0.03176649 | 0.1448 | 0.06075 |  |  |
| rs7609997 | 3 | 108,567,680 | T | 0.03176649 | 0.1448 | 0.06075 | *TRAT1* | intronic |
| rs4242185 | 5 | 174,768,731 | C | 0.03176489 | 0.1644 | 0.07477 |  |  |
| rs4731330 | 7 | 126,447,564 | C | 0.03176489 | 0.1644 | 0.07477 | *GRM8* | intronic |
| rs4745104 | 9 | 74,246,308 | C | 0.03176489 | 0.1644 | 0.07477 |  |  |
| rs4806878 | 19 | 2,846,782 | A | 0.03175287 | 0.4692 | 0.3364 | *ZNF555* | intronic |
| rs6569558 | 6 | 129,141,297 | G | 0.03169707 | 0.2089 | 0.1085 |  |  |
| rs7809531 | 7 | 90,481,699 | G | 0.03168989 | 0.3063 | 0.1887 | *CDK14* | intronic |
| rs17007529 | 2 | 29,286,670 | C | 0.0316822 | 0.3897 | 0.4764 | *C2orf71* | 3utr |
| rs11001950 | 10 | 78,729,393 | C | 0.03165196 | 0.231 | 0.1262 | *KCNMA1* | intronic |
| rs334855 | 2 | 118,099,393 | G | 0.03163611 | 0.15 | 0.257 |  |  |
| rs10121560 | 9 | 79,537,871 | G | 0.0316267 | 0.1964 | 0.09906 |  |  |
| rs4686339 | 3 | 9,340,435 | T | 0.03162076 | 0.04861 | 0.1215 |  |  |
| rs7143583 | 14 | 45,314,983 | A | 0.03161729 | 0.09155 | 0.1822 |  |  |
| rs3789775 | 6 | 52,135,494 | T | 0.03152422 | 0.3885 | 0.2617 | *MCM3* | intronic |
| rs4575402 | 13 | 90,759,420 | G | 0.03150348 | 0.4792 | 0.3868 | *LINC00559* | non-coding |
| rs6964151 | 7 | 80,553,950 | G | 0.03149904 | 0.4931 | 0.3598 |  |  |
| rs13101669 | 4 | 180,745,286 | T | 0.03148858 | 0.07241 | 0.1557 |  |  |
| rs9481842 | 6 | 118,974,798 | G | 0.03148715 | 0.1575 | 0.07009 | *CEP85L* | intronic |
| rs2095094 | 9 | 112,661,908 | A | 0.03147754 | 0.1828 | 0.08879 | *PALM2* | intronic |
| rs1730642 | 13 | 103,828,640 | T | 0.03143796 | 0.4266 | 0.4393 |  |  |
| rs7275720 | 21 | 32,484,078 | A | 0.03142285 | 0.4825 | 0.3835 | *TRIM28* | intronic |
| rs12602498 | 17 | 56,273,208 | G | 0.0313616 | 0.3036 | 0.1869 | *EPX* | intronic |
| rs7003404 | 8 | 25,871,350 | T | 0.03135422 | 0.2021 | 0.1038 | *EBF2* | intronic |
| rs10498982 | 6 | 93,348,842 | A | 0.03134045 | 0.3112 | 0.1934 |  |  |
| rs13024316 | 2 | 142,486,253 | A | 0.03132251 | 0.3298 | 0.2095 | *LRP1B* | intronic |
| rs6779450 | 3 | 72,180,966 | C | 0.03126811 | 0.1027 | 0.1963 |  |  |
| rs5755753 | 22 | 35,962,586 | G | 0.03126613 | 0.3767 | 0.4907 |  |  |
| rs6712649 | 2 | 584,523 | A | 0.03123713 | 0.2759 | 0.1636 |  |  |
| rs12611756 | 2 | 80,761,192 | C | 0.03120786 | 0.4931 | 0.3738 | *CTNNA2* | intronic |
| rs10510814 | 3 | 59,535,997 | W | 0.03114241 | 0.1781 | 0.2897 |  |  |
| rs2237553 | 7 | 86,374,533 | A | 0.03114241 | 0.1781 | 0.2897 | *GRM3* | intronic |
| rs7172348 | 15 | 60,895,223 | G | 0.03113018 | 0.3191 | 0.4486 | *RORA* | intronic |
| rs10830950 | 11 | 92,568,552 | C | 0.03109658 | 0.4433 | 0.3131 | *FAT3* | intronic |
| rs17205146 | 6 | 22,272,753 | T | 0.03107036 | 0.1631 | 0.07477 |  |  |
| rs9371388 | 6 | 150,625,469 | A | 0.03106315 | 0.3669 | 0.243 |  |  |
| rs2267206 | 22 | 33,832,148 | A | 0.03105606 | 0.09589 | 0.1869 | *LARGE* | intronic |
| rs10515044 | 17 | 51,692,084 | T | 0.03103972 | 0.479 | 0.3879 |  |  |
| rs9288570 | 2 | 222,378,994 | A | 0.03102493 | 0.3459 | 0.2243 | *EPHA4* | intronic |
| rs10762368 | 10 | 72,109,482 | G | 0.03091099 | 0.4759 | 0.3443 | *LRRC20* | intronic |
| rs10974212 | 9 | 3,937,288 | A | 0.03087544 | 0.3929 | 0.2667 | *GLIS3* | intronic |
| rs3756089 | 4 | 185,315,792 | T | 0.03086789 | 0.1821 | 0.2944 | *IRF2* | intronic |
| rs2053815 | 19 | 13,857,240 | A | 0.03080334 | 0.4965 | 0.3645 | *CCDC130* | intronic |
| rs1026078 | 11 | 45,676,823 | T | 0.03078885 | 0.4143 | 0.4528 | *CHST1* | intronic |
| rs11695180 | 2 | 115,848,781 | T | 0.03076422 | 0.3973 | 0.271 | *DPP10* | intronic |
| rs8006297 | 14 | 74,673,763 | G | 0.03076102 | 0.1286 | 0.229 |  |  |
| rs7622114 | 3 | 36,960,660 | A | 0.03075389 | 0.4276 | 0.2991 | *TRANK1* | intronic |
| rs1946936 | 8 | 70,424,271 | T | 0.03072248 | 0.5 | 0.3679 | *SULF1* | intronic |
| rs7249714 | 19 | 59,058,083 | T | 0.03065284 | 0.2482 | 0.1415 | *TRIM28* | intronic |
| rs1805870 | 8 | 91,067,602 | T | 0.03065239 | 0.3107 | 0.4387 |  |  |
| rs1620668 | 1 | 113,023,980 | G | 0.03061357 | 0.3333 | 0.4626 | *WNT2B* | intronic |
| rs6968694 | 7 | 34,217,401 | G | 0.03060566 | 0.2464 | 0.1402 |  |  |
| rs4288683 | 10 | 121,814,553 | T | 0.03059634 | 0.4101 | 0.283 |  |  |
| rs3915963 | 17 | 13,142,583 | A | 0.03053154 | 0.1404 | 0.243 |  |  |
| rs12377016 | 9 | 84,373,428 | T | 0.03050788 | 0.269 | 0.1589 | *RP11_154D17.1* | intronic |
| rs8101040 | 19 | 50,563,899 | A | 0.03049424 | 0.2069 | 0.3224 | *FLJ26850* | intronic |
| rs7582320 | 2 | 45,989,023 | G | 0.03048246 | 0.4618 | 0.3318 | *PRKCE* | intronic |
| rs17797346 | 18 | 62,033,261 | T | 0.03047937 | 0.3425 | 0.472 | *RP11_146N18.1* | intronic |
| rs6534560 | 4 | 80,688,396 | G | 0.03047222 | 0.2986 | 0.184 |  |  |
| rs2306285 | 6 | 168,699,291 | G | 0.03038435 | 0.2743 | 0.1636 | *DACT2* | intronic |
| rs16929010 | 8 | 63,395,632 | T | 0.03037776 | 0.05594 | 0.1308 | *NKAIN3* | intronic |
| rs17031463 | 2 | 106,270,503 | T | 0.03036381 | 0.0931 | 0.1822 |  |  |
| rs1532122 | 5 | 66,797,723 | T | 0.03035402 | 0.4895 | 0.3585 |  |  |
| rs12997875 | 2 | 29,434,339 | C | 0.03029785 | 0.1621 | 0.07477 | *ALK* | intronic |
| rs6061194 | 20 | 30,745,269 | G | 0.03029785 | 0.1621 | 0.07477 | *TM9SF4* | intronic |
| rs2823984 | 21 | 18,065,768 | T | 0.0302752 | 0.1747 | 0.08411 |  |  |
| rs10502001 | 11 | 102,398,593 | T | 0.03027496 | 0.137 | 0.2383 | *MMP7* | coding |
| rs6748817 | 2 | 205,258,852 | G | 0.03027496 | 0.137 | 0.2383 |  |  |
| rs10089077 | 8 | 5,348,088 | A | 0.0302108 | 0.4577 | 0.4104 |  |  |
| rs10784348 | 12 | 63,649,659 | T | 0.03021014 | 0.2122 | 0.1132 |  |  |
| rs11620231 | 13 | 41,760,947 | C | 0.03020548 | 0.4315 | 0.3037 |  |  |
| rs11950195 | 5 | 113,237,638 | A | 0.03020223 | 0.339 | 0.2196 |  |  |
| rs7017612 | 8 | 42,599,245 | C | 0.03019122 | 0.3069 | 0.1916 |  |  |
| rs2042381 | 5 | 96,188,666 | G | 0.03018729 | 0.3786 | 0.4907 |  |  |
| rs12145625 | 1 | 5,684,788 | A | 0.03018428 | 0.274 | 0.1636 |  |  |
| rs11869909 | 17 | 5,351,791 | G | 0.03017989 | 0.1818 | 0.08962 | *DHX33* | intronic |
| rs16918308 | 8 | 53,827,622 | G | 0.03016466 | 0.4113 | 0.285 |  |  |
| rs7697133 | 4 | 163,256,188 | T | 0.03016121 | 0.3429 | 0.472 |  |  |
| rs2242753 | 21 | 36,986,118 | C | 0.03002342 | 0.4928 | 0.3762 |  |  |
| rs2124432 | 2 | 129,184,166 | A | 0.03001013 | 0.2568 | 0.1495 |  |  |

**Table S3. Gene ontology and enrichment analysis of the candidate genes underlying the top 0.1% highly differentiated SNPs between the Malays from the north and south Peninsular Malaysia.**

| **Term** | **%** | **Genes** | **P Value** | **Fold Enrichment** | **Benjamini** |
| --- | --- | --- | --- | --- | --- |
| GO:0005624 membrane fraction | 2.167182663 | *ABCA1, ABCC1, ABCC4, CYP1B1, MGST2, BCAR1* | 0.004867583 | 4.096232203 | 0.377062103 |
| GO:0005626 insoluble fraction | 2.167182663 | *ABCA1, ABCC1, ABCC4, CYP1B1, MGST2, BCAR1* | 0.005813923 | 3.949763828 | 0.246328086 |
| GO:0000267 cell fraction | 2.167182663 | *ABCA1, ABCC1, ABCC4, CYP1B1, MGST2, BCAR1* | 0.019245678 | 3.059881673 | 0.46652493 |

**Table S4. Simulation analysis for GWAS before and after removing 1,666 SNPs with different minor allele present between the north and south Malays**

| **Scenario** | **Case (North)** | **Control (South)** | **λ** | **False positive (%)** | | | | **Power (%)** | | | | |
| --- | --- | --- | --- | --- | --- | --- | --- | --- | --- | --- | --- | --- |
|  |  |  |  | **Punadj** | **GC** | **Bonf** | **FDR** | **Punadj** | **GC** | **Bonf** | **FDR** |  |
| 41,400 SNPs | 300 | 300 | 3.8857 | 32.464 | 5.679 | 1.722 | 19.184 | 98.000 | 90.000 | 67.000 | 96.000 |  |
|  | 500 | 500 | 5.8070 | 41.857 | 5.558 | 4.935 | 32.060 | 99.000 | 88.000 | 88.000 | 99.000 |  |
|  | 800 | 800 | 8.7203 | 50.703 | 5.609 | 10.478 | 44.070 | 99.000 | 89.000 | 94.000 | 99.000 |  |
| 39,734 SNPs | 300 | 300 | 1.05289 | 5.207 | 5.207 | 0.131 | 0.184 | 94.000 | 94.000 | 53.000 | 68.000 |  |
|  | 500 | 500 | 1.09834 | 5.278 | 5.122 | 0.169 | 0.214 | 99.000 | 99.000 | 67.000 | 81.000 |  |
|  | 800 | 800 | 1.13641 | 5.152 | 5.152 | 0.214 | 0.242 | 100.000 | 100.000 | 84.000 | 93.000 |  |
|  | 2000 | 2000 | 1.30000 | 5.034 | 5.034 | 0.244 | 0.256 | 100.000 | 100.000 | 100.000 | 100.000 |  |
